# Clinical staff knowledge and awareness of point-of-care-testing best practices at Tygerberg Hospital, South Africa

**DOI:** 10.4102/ajlm.v9i1.853

**Published:** 2020-07-16

**Authors:** Thumeka P. Jalavu, Megan Rensburg, Rajiv Erasmus

**Affiliations:** 1 National Health Laboratory Service, Department of Chemical Pathology, Faculty Health Sciences, Stellenbosch University, Stellenbosch, South Africa

**Keywords:** POCT, near-patient testing, ISO 22870, pathology, chemical pathology

## Abstract

**Background:**

Point-of-care testing (POCT) is defined as testing done near or at the site of patient care with the goal of providing rapid information and improving patient outcomes. Point-of-care testing has many advantages and some limitations which affect its use and implementation.

**Objective:**

The aim of the audit was to determine the current practices, staff attitudes and training provided to hospital clinical staff.

**Methods:**

The audit was conducted with the use of a questionnaire containing 30 questions. One hundred and sixty questionnaires were delivered to 55 sites at Tygerberg Academic Hospital in Cape Town, South Africa, from 21 June 2016 to 15 July 2016. A total of 68 questionnaires were completed and returned (42.5% response rate).

**Results:**

Most participants were nursing staff (62/68, 91%), and the rest were medical doctors (6/68, 9%). Most participants (66/68, 97%) performed glucose testing, 16/68 (24%) performed blood gas testing and 17/68 (25%) performed urine dipstick testing. Many participants (35/68, 51%) reported having had some formal training in one or more of the tests and 25/68 (37%) reported having never had any formal training in the respective tests. Many participants (46/68, 68%) reported that they never had formal assessment of competency in performing the respective tests.

**Conclusion:**

Participants indicated a lack of adequate training in POCT and, thus, limited knowledge of quality control measures. This audit gives an indication of the current state of the POCT programme at a tertiary hospital and highlights areas where intervention is needed to improve patient care and management.

## Introduction

Point-of-care testing (POCT) is defined as testing done at or near the site of patient care, with the aim of providing rapid information and improving patient outcomes.^1^ The goal of POCT is to provide timely information regarding the patient’s condition, and to adjust management and improve the quality of care whilst avoiding medical errors.^2^

The ideal POCT programme must meet several requirements; these include organisation, supervision, written procedures, operator training and competency testing, instrument evaluation, proficiency testing, quality control, and appropriate result recording and notification.^1^ The main guidelines used to design and implement POCT programmes are the International Organization for Standardization (ISO) 22870:2016 and Clinical Laboratory Standards Institute guidelines such as POCT4,^3,4^ which provide comprehensive guidance on POCT practice. The Royal College of Pathologists of Australia has a framework for POCT in the laboratory and at POCT sites that can be adapted for use at independent sites offering POCT.^5^ These are some of the goals for a POCT programme applicable to the hospital environment.

Part of quality management may include internal quality control and external quality assurance (EQA), depending on the type of POCT device in use. Nursing staff form an essential part of the clinical team and routinely perform POCT; therefore, a regular provision of training should be provided to ensure adequate knowledge and compliance with the above requirements.^5^ Documentation, information management and record keeping are also an important part of the POCT process. Ideally, the systems should be linked to the laboratory information systems; if this is not available, the POCT team should develop programmes to ensure that this is done.^6,7^ This process includes documentation of non-conformances, and protocols on how to identify, investigate and take appropriate corrective and preventative measures. Indirect costs associated with POCT may also include training of personnel, quality control, maintenance, external quality control/proficiency testing, etc.^8,9^ Incidents of nosocomial infections related to sharing of devices between patients, as well as viral infections related to contaminated lancet holders, have been reported.^10^ These are some of the reasons why it is recommended to have a POCT committee which ensures proper training and compliance with both local and international guidelines for the implementation and management of a POCT programme.

South Africa currently has no formal national policy on POCT and training of personnel for healthcare facilities. Different hospitals implement their own programmes and it is not clear if the same protocols are followed everywhere. Point-of-care testing is widely available for use in the general and some emergency hospital units. National guidelines are necessary to inform the planning of POCT programmes, training and assessment of practitioners, or guidance on issues around quality management. Hospital and facility managers are often the main stakeholders involved in POCT programmes, without involvement of any laboratory representatives. It is against this background that different hospitals operate their POCT programmes, without national guidelines; this is also applicable to Tygerberg Academic Hospital (hereafter, Tygerberg Hospital).

Healthcare workers who use POCT at Tygerberg Hospital in Cape Town, South Africa, include approximately 550 medical staff (interns, officers, specialists, registrars, etc.) and nursing staff (approximately 2100 professional nurses, staff nurses, and nursing assistants). A variety of point-of-care tests are in use. For example, medical wards, such as the internal medicine and endocrine wards, use glucose meters and urine dipsticks on a regular basis, as do the diabetes and renal clinics. The intensive care and high care units use these POC tests as well as arterial blood gas analysers, which are benchtop POCT devices that require more skill to operate than the other two tests and are often performed by medical staff, professional nurses or technicians, when available.

This study audited current practices and training provided to hospital personnel who regularly use POCT at Tygerberg Hospital, to determine whether collaboration is necessary between the laboratory and the hospital management team responsible for the current POCT instruments used. The audit aimed to determine the current training provided to clinical staff about the use of POCT devices, investigate staff practices and attitudes towards POCT, and to ascertain the general level of training and knowledge of quality control in the practice of POCT. The information gathered will help to give feedback to the stakeholders on areas that can be improved in the current POCT programme. Such areas include staff training, competency assessment, and regular refresher courses on both the theory and the practical aspects of POCT. The stakeholders include hospital administration, nursing staff, doctors, clinical technologists, laboratory technologists and pathologists of the relevant disciplines. The audit was not requested by the hospital; it was laboratory initiated.

## Methods

### Ethical consideration

Ethics approval was obtained from the University of Stellenbosch health research ethics committee (Reference: S15/11/269). Written permission was also obtained from the hospital management to conduct the audit in the hospital medical wards and outpatient clinics. Participants were given information leaflets and signed informed consent forms after agreeing to participate in the study.

### Study design

This study is a descriptive, cross-sectional audit conducted with the use of a questionnaire containing 30 questions (Supplementary Document 1). A questionnaire was chosen as the most ideal and feasible format for conducting this qualitative audit. The questions were designed to cover the important aspects, based on the ISO 22870 guideline.

### Questionnaire

The questionnaire was developed by the primary investigator, with the input and supervision of the co-investigators, based on a thorough literature review. This initial draft was further adapted to include the relevant sections from ISO 22870 in order to incorporate quality management aspects. Questions included general knowledge about POCT at the different sites, theoretical knowledge around POCT, formal training and competency assessment of POCT operators, quality control measures and current perceptions of operators on their respective tests. Formal training was defined as a lecture-type session lasting approximately 1 hour, including a practical demonstration and practice in the use of the specific POCT device. Questions were grouped in sections, with a few confirmatory questions which did not follow a specific order. Routine performance of tests was assessed by asking about the frequency of tests performed per week; that is, participants were asked to indicate the average number of times they performed each test applicable to them (more than one test could be selected from a table). The questionnaire was validated at two locations and with colleagues within the department to ensure the questions were understandable prior to distribution of questionnaires.

### Setting and tests included

This study was conducted at Tygerberg Academic Hospital, a tertiary hospital with an inpatient capacity of 1384 beds situated in the northern suburbs of Cape Town, Western Cape, South Africa. The hospital serves a community of approximately 3.6 million from the public health system. Three point-of-care tests – blood glucose, blood gases and urinalysis – were chosen for the audit, because they are performed routinely throughout the hospital and fall under the expertise of the study team. All POCT devices used in the hospital are from Roche ACCU-CHEK® (Roche Diabetes Care GmBH, Mannheim, Germany). Other non-chemistry point-of-care tests, such as those for HIV haemoglobin, were not included in the audit as they fall outside the scope of the investigators’ expertise.

### Data collection and analysis

An average of three questionnaires was delivered to 55 sites in the hospital, comprising wards, emergency units and outpatient clinics, between 21 June 2016 and 15 July 2016. A few sites only accepted one or two questionnaires, because the nursing managers could not identify anyone else who was suitable to participate, so the total number of questionnaires delivered was 160. Site inclusion criteria included any hospital site that routinely uses POCT as part of patient management. This included all general medical and surgical wards, emergency units, intensive care units and high care units.

The psychiatric and orthopaedic wards and clinics were excluded, because of the low likelihood for use of POCT at these sites.

Ward managers were approached to help with selection of suitable nursing staff to complete the questionnaires. We expected professional nurses, staff nurses and nursing assistants to form the majority of participants from the nursing side. We also expected interns, medical officers and registrars to form the majority of participants from the medical staff. This expectation was based both on their respective clinical duties and on their close involvement in daily patient care as medical doctors without the competing managerial duties applicable to senior and higher rank medical consultants and specialist doctors. Students were excluded from the study because they were not employed by the hospital and were at different levels of study.

Participants were given an option to complete the questionnaire at the time of delivery or to complete it in their own time when not busy with core duties. All participants were encouraged to complete the questionnaire as comprehensively as possible and all those who delayed in completing the questionnaire were given a second or third chance to do so.

Information from the questionnaires was captured on Microsoft Excel 2016 version 16.0 (Microsoft, Redmond, Washington, United States), which was used to calculate basic descriptive statistics for the data. In the analyses, each ward or clinic included in the study counted as a single site. Missing data included questionnaires that were returned uncompleted and those which were completed only in part. The latter group was included in the data analysis as most of them had completed over 80% of the questions. Missing data were also taken into account when specific questions were reported. Responses are reported as percentages of the final sample size. Each question analysed includes an indication of the percentages of those who did not answer the specific question (Tables 1 and 2). No further adjustments were made to compensate for missing data.

**TABLE 1:** Participant characteristics, ranks and clinical experience of Tygerberg Hospital, Cape Town South Africa, 2016.

Participant characteristics	Frequency	%
**Participant qualific ations (*n* = 68)**		
Medical doctor	6/68	9
Nurse	62/68	91
**Medical doctor rank (*n* = 6)**		
Registrar	5/6	83
Medical officer	1/6	17
Intern	0/6	0
**Nursing staff rank (*n* = 62)**		
Registered professional nurse	27/62	44
Staff nurse	9/62	15
Auxiliary/assistant nurse	8/62	13
Enrolled nurse	7/62	11
Unspecified rank	11/62	18
**Clinical experience (*n* = 68)**		
1 to 5 years	23/68	34
6 to 10 years	10/68	15
11 to 15 years	2/68	3
16 to 20 years	1/68	1
More than 20 years	13/68	19
Unspecified	19/68	28
**Frequency of POCT device use per week (*n* = 68)**		
Less than 3 times	8/68	12
Three to five times	4/68	6
More than five times	52/68	76
Invalid responses	4/68	6

POCT, point-of-care testing.

**TABLE 2:** Awareness and knowledge of point-of-care best practices amongst healthcare workers at Tygerberg Hospital, Cape Town, South Africa, June-July 2016.

Quality measure	*n*	%
**POCT distribution among participants**		
Glucose testing	66/68	97
Blood gas	16/68	24
Urine dipsticks	17/68	25
Use of 2 or more tests	52/68	76
**Competency assessment**		
Yes	8/68	12
No assessment offered	22/68	32
Demonstration	8/68	12
Last assessed in nursing college	24/68	35
Invalid and unanswered	6/68	9
**Report of formal training**		
Formal training	35/68	51
No formal training	25/68	37
Unanswered	8/68	12
**Time since formal training**		
Within 6 months	9/35	26
1 year prior	4/35	11
More than 2 years prior	21/35	60
Unanswered	1/35	3
**Participant subjective need for training**		
Training desired	45/68	66
Training not desired	17/68	25
Unanswered	6/68	9
**Knowledge of POCT device validation**		
Validation by clinical engineering	20/68	29
Validation done by the ward	3/68	4
No knowledge of validation	36/68	53
Invalid and unanswered	8/68	12
Validation not done	1/68	1
**Subjective rating of the importance of POCT device maintenance (scores)**		
Score 1: not important	1/68	1
Score 2: maybe important	1/68	1
Score 3: Neutral	9/68	13
Score 4: important	16/68	24
Score 5: very important	38/68	56
Unanswered	3/68	4
**Reported necessity of POCT by participants**		
Yes, it is very important for patient care	52/68	76
Most of the time it helps to manage our patients	6/68	9
I’m not sure it makes a difference	1/68	1.5
It does not really help in our ward/clinic	1/68	1.5
No selection/answer given/contradictory selection	8/68	12
**Knowledge of person in charge/manager of device maintenance and replacement**		
Aware	42/68	62
Not aware	14/68	21
Unsure	10/68	15
Unanswered	2/68	3
**Requirement for Operator ID to use/operate the blood gas instrument**		
Yes, access is by operator specific ID only	9/68	13
Yes, but IDs are shared by users sometimes No, there is no login ID required to perform tests	8/68 0/68	12 0
I do not operate the blood analyser	21/68	31
Unanswered	30/68	44
**Participation in EQA schemes**		
Yes, we have an EQA programme	6/68	9
I am not aware of such a programme	37/68	54
I don’t think it is needed	5/68	7
I don’t know what that is, or how it is done	15/68	22
Unanswered	5/68	7
**Availability of written protocols for operation of POCT devices**		
Yes	49/68	72
Not available	13/68	19
Unanswered	6/68	9
**Available protocols for specific indications according to participants**		
Very high glucose	68/68	100
Expired urine dipsticks	46/68	68
Expired glucose strips	44/68	65
Changing reagents on the blood gas instrument	32/68	47
**Access to manufacturer manuals**		
Always have access	34/68	50
Unsure where manuals are kept	16/68	24
No access	11/68	16
Unanswered	7/68	10
**Appropriate handling of strips with prolonged exposure to air**		
Close container and continue use strips	12/68	18
Document and report to ward manager and use new strips	44/68	65
No action required	3/68	4
Other	7/68	10
Unanswered	2/68	3
**What is important before performing a POCT in the ward**		
Patient preparation	47/68	69
Confirmation of results	9/68	13
Documenting the last meal	3/68	4
Following supervisors’ example	1/68	1
Invalid/no answer	8/68	12
**Which method to use if a patient has severe dehydration and shock**		
Glucose meter	23/68	34
Blood gas analyser	11/68	16
Laboratory glucose	25/68	37
Other (unanswered and more than 1 selection)	9/68	13
**Does the blood gas meter always give accurate results like the laboratory?**		
Yes	7/68	10
Most of the time	38/68	56
Sometimes	10/68	15
Unsure	11/68	16
Unanswered	2/68	3
**Importance of record keeping**		
Recording system used	21/68	31
No need for record keeping	19/68	28
No knowledge of recording system	24/68	35
Invalid/unanswered	4/68	6
**What to do/who to call for faulty devices**		
Specific person named/workshop	30/68	44
Sister in charge	28/68	41
Use a different one or send samples to the lab	1/68	1
Borrow from another ward/clinic	5/68	7
Invalid or no response	4/68	6
**Availability and use of a recording system for POCT results (excluding patient files)**		
Recording system available	21/68	31
Recording system not required	19/68	28
Unaware of a recording system	11/68	16
Other system given by participant	13/68	19
Invalid response or unanswered question	4/68	6

POCT, point-of-care testing; ID, identification; EQA, external quality assurance.

## Results

### Study participants and tests administered

Out of the 160 questionnaires delivered, 68 were returned completed (42.5% response rate) (Table 1). Most participants (66/68, 97%) performed glucose monitoring, 16/68 (24%) performed blood gas testing, and 17/68 (25%) performed urine dipstick testing (Table 2). A total of 52/68 (76%) performed one or more of the tests more than five times per week, mainly glucose, followed by urine dipsticks and blood gas analysis (Table 1).

### Knowledge and awareness of point-of-care-testing best practices

Although a majority (35/68, 51%) indicated that they had formal training, many indicated that they had not (25/68, 38%) (Table 2). Those participants who indicated that they had knowledge of device validation, reported that this was performed by either the clinical engineering department or the ward. Most respondents (57/68, 78%) indicated that POCT is necessary in their respective wards or clinics. Most of the staff (42/68, 62%) indicated that they knew who was managing POCT in their respective locations.

The present study found that 31% (*n* = 21) of participants indicated they used a recording system in addition to patient files, 28% (*n* = 19) indicated they did not require use of a recording system, and 16% (*n* = 11) were unaware of a recording system in their ward or clinic. Nineteen percent (*n* = 13) of participants indicated using other means of record control, mostly involving duplicating of result entries in the nursing notes as well as the designated charts on patient files; one participant indicated that doctors enter POCT results on their computer for future reference. Six percent (*n* = 4) of participants selected more than one of the four answer options, thus providing conflicting responses.

When asked about the important step(s) before performing a point-of-care test, 47/68 (69%) correctly indicated that patient preparation was vital, whilst 9/68 (13%) indicated that confirmation of results was an important first step. A large proportion (25/68, 37%) indicated that the laboratory method was more accurate for glucose measurement in a patient with dehydration or shock, whilst 23/68 (34%) felt the glucose meter was as accurate as the laboratory; a further 11/68 (16%) indicated that the blood gas measurement is the most reliable if a patient is dehydrated. Record keeping of test results was another parameter used as a marker of quality management in POCT; 21/68 (31%) of participants said they used a recording system, 19/68 (28%) felt there was no need for it, and 24/68 (35%) either did not know if there was a recording system or selected other forms of a recording system. More than half of the participants (38/68, 56%) viewed POCT as being an important part of patient management (Table 2).

## Discussion

Most respondents to this audit of the use of POCT by clinical staff at a South African tertiary hospital found that POCT was a vital part of patient care; this is important, as it is likely to ensure that the staff is open to learning and keeping up to date with new information and practices. The second main observation was that there is a lack of formal training of hospital staff in the practice of POCT, and most of the participants indicated that they needed formal training in POCT. This is an important issue which requires consideration by stakeholders as it may impact patient outcomes and improve staff confidence in performing the tests.^11^ Staff confidence requires formal skills training and competency testing in order to minimise the risk of errors.^12^ Errors can be attributed to several underlying reasons, including poor technique, abnormal haematocrit, failure to adhere to the correct procedure, and presence of interfering substances. For example, the POCT devices used in the hospital are Roche ACCU-CHEK® devices, which are known to be prone to galactose, ascorbic acid and ceftriaxone interference, and which may deliver false high or -low glucose results in the presence of interference.^13^ The package insert also states that the use of the glucose meter is not advised in patients with peripheral vascular disease or with dehydration from several causes. Without theoretical knowledge relevant to the test performed, the clinical personnel are at a disadvantage and are not fully equipped to perform these tests. All persons involved in POCT should be aware of potential interferences, why patient preparation is important, and the concepts of accuracy and precision. Such knowledge requires training by experts in the field, such as laboratory professionals who would provide valuable input in the training of clinical personnel.

The participants showed a limited awareness of quality control procedures, such as POCT device validation and EQA. This was indicated by the high number of participants (54%, *n* = 37) who were not aware of any EQA involvement in their ward or clinic and the 53% (*n* = 36) who did not know if any device validation or verification was performed prior to the use of new POCT devices in their wards or clinics. This is related to the lack of training in POCT basics and principles. The purpose of POCT device validation, internal quality control and EQA is to ensure that the results obtained are of a good quality and give confidence to the clinician who will initiate or change the treatment of the patient based on the result obtained from a POCT device. Laboratories are required to participate in internal quality control and EQA activities in order to be accredited to international standards. Point-of-care testing programmes also benefit from such quality control measures, as this would allow them to compare with other POCT sites and allow early identification of non-conformances. International guidelines, such as ISO 22870:2016 and (CLIA) POCT04, recommend operator training in both the theory and the practice of internal quality control of POCT devices.^3^

Some countries, such as Australia and New Zealand, have local guidelines on the use and implementation of POCT based on both national and international recommendations.^6,14^ When testing for glucose, theoretical knowledge is required in order for the tester to be aware of factors such as haematocrit levels, systemic shock oxygenation status and exposure of strips to humidity, which can reduce the shelf-life of the strips.^15,16^ A low haematocrit level (< 30% – 35%) may lead to overestimation of glucose, whilst a haematocrit above 45% may lead to underestimation of glucose results by some POCT devices. Some of the above factors have predictable effects, such as overestimation or underestimation of tests such as glucose, or to the delivery of false-positive dipstick results because of exposure of the strips to humidity. Exposure of glucose meter reagent strips to humidity does not have a predictable effect of over- or underestimation of glucose results, unlike the aforementioned examples. Some POCT devices for glucose have been shown to have poor accuracy at critical glucose levels (critically high: > 33.3 mmol/L; critically low: < 2.2 mmol/L) and to have significant bias compared to other devices and the central laboratory method.^17^ It is therefore important for POCT operators to know when to question a POCT device result and to confirm with the central laboratory method. Some studies have also shown that POCT in high-risk patient groups, such as those patients in either adult or paediatric intensive care units, can lead to misdiagnosis.^16,18^ This requires staff to be very knowledgeable about potential sources of error, dealing with critical values and the use of protocols to guide POC test use in these settings.

Point-of-care testing has become an integral part of healthcare in both the primary care and the hospital setting.^1^ With the increasing use of POCT, there is also an increasing need to adopt and practise global principles to avoid medical errors and ensure patient safety.^2^ Potential disadvantages of POCT include insufficient validation of trained and certified operators, insufficient supervision, limited understanding of quality control testing, little or no security of patient test results and quality control data and limited connectivity of POCT devices.^2,12^ This audit sought to evaluate the current state of POCT practice at Tygerberg Hospital by focusing on the most widely-available and commonly-used tests in the hospital. In agreement with international practices in the hospital setting,^5,12^ nurses form the bulk of the POCT operators in this study. However, Nnakenyi et al. showed different findings in their audit of 5 hospitals in Nigeria. Their study had 40% physicians, 32% nurses and 27% technologists, with a total of 98 participants across all five hospitals.^19^ The study above is comparable to the present study in terms of sample size and recorded a good response rate from doctors. Our study was targeted at clinical staff, namely, nurses and doctors only. Great effort was made to recruit doctors in this study; the poor response rate from the doctors in this study may indicate the low level of interest of doctors in POCT. This finding supports the recommendation by some to keep POCT programmes under the control of the laboratory. This would mean that the Head of Chemical Pathology or the principal chemical pathologist becomes the chair of the POCT committee in the hospital, and she or he would be directly involved in the decision making and running of the programme as recommended by international guidelines.

In many hospitals in sub-Saharan Africa, POCT is performed by clinical staff because of the limited availability of medical technologists, who are primarily employed in core laboratories with limited numbers, if any, involved in hospital POCT. Clinical staff are therefore at the forefront of hospital POCT in sub-Saharan Africa and a good source of information about the practices, successes, and limitations of hospital POCT in this setting.

A study conducted in Nigeria included only doctors, which provided a different perspective but limits a direct comparison between the different African studies.^20^ This study used an interviewer-administered questionnaire on doctors at two different hospitals in Nigeria. The sample selection method was not explained clearly; the response from the two hospitals seems to have been 22% for one and 32% for the other. There is no specific mention of how the selection process was conducted and the rationale behind the exclusion of nursing staff in their study. Our study, by contrast, mainly included nursing staff who are the main operators of POCT in the hospital setting. The questionnaire was designed to obtain information, to identify current gaps in the system and to find solutions that may be easy to implement. Some questions were asked to elicit information about record keeping and maintaining a clear paper trail. This has been shown to be a limitation of many POCT programmes where devices do not have connectivity to the laboratory system and rely only on the manual transcription of results.^21^

There were varied responses to these questions in this study, and this may point to the lack of a formalised system for recording POCT results separate from the entries in patient files.

Focus group discussions have been conducted amongst personnel to determine their perception and operational impact of POCT on clinical duties in parts of rural Australia and Uganda (23,24).^22,23^ Most participants of these focus groups indicated that they valued POCT, but were dissatisfied with the implementation and their exclusion from the planning process. In our setting, the use of focus groups was not feasible, because of staff shortages and the limited time available to engage with the nursing staff whilst they concentrated on their clinical duties. The present study found that the majority of participants (76%, *n* = 52) also valued POCT and regarded it as being an important component of patient care in their environment. Many (66%, *n* = 45) indicated that they would want formal training in POCT, 25% (*n* = 17) indicated no desire to have training in POCT, and 9% (*n* = 6) did not answer the question. The questionnaire did not specifically include questions about views on implementation or involvement of participants in the planning of the POCT programme in the hospital. The focus was primarily on the practice of POCT, knowledge of POCT theory, and perceptions of participants regarding POCT in the wards and clinics where POCT programmes are already implemented.

The findings of the study are mainly applicable to Tygerberg Hospital and to other tertiary hospitals that do not currently have a POCT training programme, and where the central laboratory is not involved in POCT. These findings may not apply to other hospitals in South Africa who have a different POCT management system. The training of nurses should be explored in other institutions in South Africa to give a comprehensive picture of the POCT programmes in local hospitals. This should be followed by the development of training programmes and regular re-training to ensure that clinical personnel keep their knowledge and skills up to date. At the time this audit was conducted, there were no national guidelines or policies guiding the practice of POCT in South African hospitals. There is limited published information on current practices in POCT in South Africa and within the rest of the African continent. Many of the studies available in Africa have looked at implementation of specific POCT instruments and clinical outcomes. These studies do not primarily look at the availability of local guidelines and training of personnel on POCT and therefore cannot be compared directly with the current study. Compared with other POCT programmes, such as HIV-POCT, general POC biochemistry tests have been around for much longer. A collaborative study of Zambia and South Africa on HIV-POCT found that intensive training, supervision and robust quality assurance mechanisms were required to optimise community HIV-POCT.^24^ A similar approach can be applied to other POCT programmes to improve their quality.

This audit is the first of its nature to be conducted and reported in South Africa. It will provide a basis for the laboratory and hospital to determine the need for collaborative training of clinical staff.

### Limitations

The limitations of the study include the small number of questionnaires sent, which was estimated based on the knowledge that not all staff in the ward perform POCT and that those who do are usually busy with clinical duties and we did not want to distract them from service delivery. The response rate was quite low overall; in some sites, available staff members were busy when questionnaires were distributed and even upon follow-up, they still did not have time to complete them.

This applied to both nursing staff and the mid-level/junior medical staff.

The questionnaire did not focus on the views of participants regarding planning and implementation of POCT programmes in the hospital, this information would have been valuable and used to gauge the general attitude of participants in being directly involved in the planning and implementation of POCT in the hospital. Some questions may not have been clear or explicit enough for participants to provide accurate feedback; even though the questionnaire was piloted with nursing staff and medical doctors, some participants may still have found some questions unclear.

### Recommendations

We recommend the introduction of training and certification programmes for point-of-care test operators in keeping with international guidelines. We also recommend that a POCT coordinator be appointed to lead the current programmes with the assistance of a dedicated POCT team in the hospital, as well as the involvement of the clinical laboratory for the continuous evaluation and improvement of the current programme.

### Conclusion

This audit found that a significant percentage of the participants did not receive adequate training in POCT and had very limited knowledge of quality control measures. This audit gives an indication of the current state of the POCT programme in the hospital and highlights areas where intervention is most needed to improve patient care. Current guidelines recommend that hospital personnel have basic knowledge and skills to perform routine POCT. Appropriate implementation of a POCT service requires focus on all aspects, including staff training and quality assurance. This information is also important to inform the Department of Health of the need to consider implementing guidelines and policies on POCT in all health facilities in South Africa.
